# Monitoring and Evaluation of Late Functional Outcome in Post-treatment Follow-Up in Clinical Routine Setting

**DOI:** 10.3389/fonc.2019.00700

**Published:** 2019-07-30

**Authors:** Veit Zebralla, Sylvia Meuret, Susanne Wiegand

**Affiliations:** Clinic of Otolaryngology, Head and Neck Surgery, University Hospital Leipzig, Leipzig, Germany

**Keywords:** head neck cancer, PRO, aftercare, follow-up, survivorship, functional impairment

## Abstract

Patients treated for head and neck cancer (HNC) often suffer from severe and visible loss of function as the cancer itself and the side effects of aggressive treatments have the potential to severely affect quality of life. Therefore, the aim of follow-up is not only the early detection of potentially curable recurrences and second primary tumors but also the diagnosis and rehabilitation of functional impairments. Clear guidelines determining the frequency of follow-up visits are missing, and the impact of follow-up visits on patient's prognosis is unclear. An intensive post-treatment follow-up is needed to detect functional impairments and to initiate their treatment. The aim is an optimal rehabilitation of the patients. This article focusses on goals of aftercare treatment and describes the spectrum of long-term sequelae, and the impact of Patient Reported Outcome (PRO) instruments of which three will be introduced.

## Aftercare in Head and Neck Cancer Patients

A systematic definition of the aftercare follow-up does not yet exist. Most head and neck oncologists perform the aftercare in an individual way. The goals in head and neck cancer aftercare are different from most other tumor entities. Orientating on the recommendations of the American Cancer Society (1) and the United Kingdom National Multidisciplinary Guidelines (2) the goals of aftercare will be defined in the following section.

The first goal is surveillance for recurrence of the cancer and detection of second primary tumors. Until now it is unclear whether surveillance provides any survival advantage in HNC (2). The survival rate of patients with recurrent disease is very poor, so screening for recurrent cancer is extremely important (3). Follow-up usually includes physical examination along with radiological imaging in the case of tumor suspicion (4). A high rate of HNC survivors (about 23%) develop a second primary cancer, mostly in the head and neck area, lung, and esophagus (5). Early detection of secondary cancer can lead to better prognosis and reduces treatment associated comorbidity (6).

Detection and treatment of therapy associated side-effects is another goal of follow-up in clinical routine setting. Early monitoring for treatment-related side-effects like acute complications of surgery and/or (chemo)radiation and long-term sequelae like fibrosis and nerve palsy is imperative. The additional screening for negative psychological effects of the disease and its treatment is also very important as psychological challenges are often underestimated. Up to 80% of all HNC patients reported social disruption and about 70% depressive symptoms in a study published in 2012 (7). Patients with depression have a higher mortality from cancer and non-cancer specific diseases (8, 9). The monitoring of physical and psychological issues should be equally considered, to realize the aftercare treatment in a holistic way. This goal becomes more and more relevant due to new therapeutic strategies and a younger population of HNC patients through an increasing number of HPV related oropharyngeal carcinoma and expected longer survival times (10, 11). It has to be discussed if the usual follow-up of 5 years is long enough.

Health promotion should be a further aim of good aftercare. The clinician should inform patients about support groups to reveal resources and counsel them to avoid tobacco and alcohol. Additionally, the clinician should explain the patients the positive effects of nutrition, activity, and healthy behavior (1, 2).

In conclusion, the head and neck surgeon is the coordinator of the oncological aftercare, who has to manage the communication to other health providers, include other stakeholders and professions and monitors the patient in a global way. A relevant problem is the lack of evident data in aftercare research.

## Special Aspects and Late Term Functional Impairments in HNC

Patients with HNC suffer from severe and often visible functional impairment due to the tumor itself and its therapy. As explained, these impairments include physical and psychological issues. In contrast to other tumor entities, HNC impairs all basic functions of daily life, especially in advanced tumor stages. Swallowing, eating, smelling, voice, speech, and breathing can be directly affected by the tumor and its therapy. Unique to HNC, the effects of treatment may be highly visible, altering appearance, and the confidence to return to social activities (12).

### Swallowing and Nutrition

The effects of therapy of HNC on swallowing and nutrition are well-described in literature (13–16). All therapy modalities have the potential to induce late functional swallowing impairment. While surgery often causes problems like fistula, diverticula, and stenosis in early and midterm follow-up, chemoradiation often leads to negative sequelae in the long-term follow-up. The functional outcomes after surgical resection and reconstruction can vary considerably with tumor stage, localization, type of reconstruction, and surgical expertise. After major surgery of HNC functional impairment is common and is associated with poorer swallowing-related quality of life (17). Radiation-induced fibrosis can result in scarring and stenosis of the swallowing passage, increasing the likelihood of penetration and/or aspiration and the need for a feeding tube (18). Other common problems are xerostomia, osteoradionecrosis, and trismus (19, 20). A tracheostomy or laryngectomy may be required for airway management, further compounding swallowing difficulties. The screening and monitoring for these problems using questionnaires like EAT-10 and the MD Anderson Dysphagia Inventory (MDADI) are highly recommended in every tumor aftercare consultation in the opinion of the authors. The gold standard of swallowing function assessment is an instrumental test such as videofluoroscopy (VF) or the fiberoptic endoscopic evaluation of swallowing (FEES) (21). These diagnostic tests are used to identify the presence of penetration and/or aspiration and the pathophysiology of dysphagia (22). Speech-language pathologist and dietary adviser should support the learning and use of swallowing maneuvers and nutrition. If swallowing problems are caused by a bad dental state or by osteonecrosis of the jaw, a dentist should be included in the follow-up (23).

### Voice and Breathing

Loss of voice and changed speech are severe problems in many patients who underwent therapy for laryngeal cancer. Superior objective voice outcomes have been reported for patients undergoing a partial laryngectomy when compared to total laryngectomy (24). Conversely, patients self-report higher satisfaction with their voice following laryngectomy than those having a partial laryngectomy (25, 26). Therapy of the oral cavity and pharynx also can lead to problems in voice, speech, and articulation in short and long term follow-up (27). There are enormous effects on the individual caused by social isolation: not returning to work and higher rates of depression and decreased quality of life. The Voice Handicap Index (VHI) is an easy instrument to gather information on the patient's perception of their voice (28). The Carcinologic Handicap Index was developed and psychometrically tested in 2017, covering a broader range of symptoms, using the VHI as its basis (29). The patient should get access to a speech and language pathologist to have the possibility of a good functional outcome.

Breathing is another important aspect in HNC follow-up. Tracheostomy tubes require routine changing which may be performed by the patient or nurse with special training. Our own experiences show, that patients often have problems with closed tracheal cannula, dyspnea, and tracheal infections and the hospitalization rate due to breathing problems is high. Tracheitis and withdrawal from social activities like communal meal are commonly reported (30). Frequent long term problems could be laryngitis with dyspnea, progredient aspiration and aspiration pneumonia, subglottic tracheal stenosis after tracheotomy, progredient internal disease like pulmonary fibrosis and cardiovascular problems (31, 32).

### Musculoskeletal Effects

Spinal accessory nerve injury is a common complication of neck dissection and leads to reduced shoulder mobility and pain (33). Other nerval complications include impaired arm mobility (brachial plexus) and facial muscle mobility (facial nerve). The late effects due to radiotherapy and surgery like neuropathy and lymphedema, strictures and scars, dystonia, and trismus can also result in reduced head and neck motion often combined with pain. To observe and treat those late effects in the long term follow-up a complete anamnesis and physical examination is needed. The collaboration with a physiotherapist is essential to reduce these late term side effects.

### Psychosocial Effects

Patients who underwent therapy of HNC are highly affected by psychological co-morbidity (34). The fear of recurrence and dying are common reported issues. The challenges the patient must deal with are influencing all areas of social life: body image and self-image, sexual function and relationship, social role, employment and return to work, depression and depressive symptoms, worry and anxiety (35, 36). The assessment of these challenges is recommended, especially because the number of untreated psychological co-morbidity like distress is high (37). Instruments to assess psychological and psychosocial effects are the commonly used Hospital Anxiety and Depression Score (HADS) (38) and the NCCN Distress Thermometer (39). The patient should refer for psychosocial care to a specialized section.

## Instruments For Clinical Long-Term Follow-Up

HNC patients suffer from unmet needs and a high rate of under-reported symptoms (40, 41). In order to describe and document all relevant findings, new instruments were developed in cancer aftercare. Routine anamnesis and clinical examination are necessary and the contact patient to physician is indispensable. But the literature also shows, that Patient Reported Outcomes (PRO) may address these problems. A PRO is defined as “…any report of the status of a patient's health condition that comes directly from the patient without interpretation of the patient's response by a clinician or anyone else” (42). While in clinical trials use of patient reported outcome measurements (PROM) is established, use in clinical routine setting is not regularly implemented. However, PROMs have a lot of possible benefits and barriers that are discussed in literature (43).

Benefits of PROM:

- Benefits for patient to physician communication (more holistic patient and physician communication)
- Identification of unreported side-effects and functional impairments
- Positive value on shared decision-making process
- Better quality of life scores compared to the routine care group
- Higher satisfaction with tumor aftercare and follow-up examination

Barriers of PROM implementation Include:

- The questions are irrelevant for the patient
- Interpretability of scores measured
- Need of time and cost factors

To reduce the barriers and reach a higher acceptance in stakeholders of the health system electronical Patient Reported Outcome Measurements were developed. A trial comparing paper completion to an electronic version of the EORTC QLQ- H&N35 did not find a difference regarding response or interpretation of the items (44). In the field of HNC, electronic PROMs are rarely implemented and benefits and barriers are not described in daily clinical practice very well (45). The software tools herein described are examples for the successful implementation of ePROMs in head and neck cancer centers.

### OncoQuest

In 2006 “OncoQuest” was implemented in clinical routine setting in Amsterdam. This system monitors health-related quality of life using the EORTC QLQ-C30 and QLQ-HN35 questionnaires and the Hospital Anxiety and Depression Scale (HADS) (46). In sum, 79 questions were asked to the participant. This system is an “in clinic system” which means that it is used only in the clinic itself. The physician has the possibility to see all answers and the calculated values in real time on a screen. In a trial published in 2017, the authors described a good usage rate, but they also described barriers for using their system (45). Patients should use the “OncoQuest” system in every follow-up visit and the authors of the study also described differences between “ever users” and “never users.” The most common reported issues in “never users” were lack of time, no questions regarding supportive care and absence of symptom change.

### OncoFunction

The ePRO Measurement instrument “OncoFunction” is also an “in clinic system.” It is based on the International classification of Function (ICF) of the World Health Organization (WHO). The final questionnaire was published in 2017 and is recommended by the German Cancer Society (47). The system also has two parts: the patient's and the physician's questionnaire. The data entry is done by the patients themselves using tablets. The physician sees the PRO in real time on a screen. Using the physician's questionnaire leads to a complete and comparable documentation after clinical examination of the patient. The implementation of the system started in 2013. The feasibility and the implementation was first described in 2016 (48). There was no difference in UICC state and tumor localization between users of the system and patients not using OncoFunction in the aftercare consultation. An increased satisfaction and good acceptance of the program was described in a patient-based survey.

### OncoKompas

“OncoKompas” is an eHealth Application specifically designed for use in the clinic (49). It is a web based self-management tool which allows the patient to complete information at their own chosen frequency. A digital copy of the “diary” could be shown to the health care professional. This seems to be a more patient centered approach which simplifies the contact to the health care professionals also in case of a problem. The barriers to implement this progressive tool were high. Only 31% of the eligible hospitals in The Netherlands implemented the tool for the trial. The data of patient's acceptance are not published yet.

These three examples show a excerpt of developed PRO measurement systems in the past years. Other publications describe programs such as Kaiku® using an ePROM in HNC patients (50, 51). Most publications investigating the implementation and acceptance of PROM and ePROM in HNC patients are based on short term follow-up but only few focuses on the long-term experience of implementation of a PRO system. There is a need to get more information about difficulties and barriers implementing those systems in routine clinical setting.

## Perspective and Requirements

The screening and monitoring of late functional outcome have become more and more relevant in recent years. The increasing number of trials which declare the functional outcome after therapy as primary endpoint shows the importance of this field. Due to the fact that more and more younger patients are suffering from HNC, there is a need of individual aftercare especially in long-term follow-up.

In 2017, Basch et al. showed a higher survival rate in cancer patients using a web-based PRO instrument between their regular visits (52). This study excluded HNC patients and included only patients with metastatic disease. The demonstration of better survival rates using a PRO instrument compared to the usual care group was new and important. Whether these findings are transferable to the curative treatment of HNC patients or affect the contact frequency to physicians in hospital is still unclear. A review on the value of the routine use of patient-reported outcome measures identified just five trials (no HNC patients) and reported inconclusive findings (53).

Beside the patient-related aspects, administrative aspects will be relevant in the future. Most of the PRO instruments (not only in HNC) are separate stand-alone systems. The development of such systems is easier, and the implementation in a single institution can easily be realized. To bring these systems into comprehensive daily clinical use, links to the local electronic health record is needed (54). The development and integration of PRO assessment into the electronic health record also will take resources from many stakeholders, clinicians, researchers, programmers, health care system administrators, and patients (55).

HNC patients have special needs (mostly not comparable to other entities) and the problem of “negative selection” exists. Patients who cannot or are not be willing to answer PRO questionnaires will need tailored aftercare concepts.

So far, the monitoring of late functional outcome in the clinical routine setting for HNC patients is not comparable between clinics and there is no gold standard. It could be anticipated that in future an increasing number of departments treating HNC patients will integrate PRO assessments in the clinical aftercare and routine practice. The described development of web applications seems to be the next step in monitoring patients also in homecare. The benefit of individual aftercare which is not strictly orientated on time schedules is obvious. The need for intervention maybe detected earlier and the barrier to engage a medical health professional could be lowered. For this purpose, more research in the field of head and neck cancer aftercare is needed and more information about the optimal kind of PRO instruments and the required data must be collected.

## Conclusion

Patients who underwent therapy of HNC often suffer from highly visible and significant functional impairment. Patient Reported Outcome instruments can lead to better aftercare results and higher patient satisfaction. Beside all positive aspects and advantages, there are many barriers existing in implementing such systems. Nevertheless, PRO instruments will be implemented in an increasing number of oncological departments to collect complete and comparable data. In the future, applications will increasingly be available for homecare use.

## Author Contributions

All authors listed have made a substantial, direct and intellectual contribution to the work, and approved it for publication.

### Conflict of Interest Statement

The authors declare that the research was conducted in the absence of any commercial or financial relationships that could be construed as a potential conflict of interest.

## References

[1] Cohen EE LaMonte SJ Erb NL Beckman KL Sadeghi N Hutcheson KA. American Cancer Society head and neck cancer survivorship care guideline. CA Cancer J Clin. (2016) 66:203–39. 10.3322/caac.21343 27002678

[2] Simo R Homer J Clarke P Mackenzie K Paleri V Pracy P. Follow-up after treatment for head and neck cancer: United Kingdom National Multidisciplinary Guidelines. J Laryngol Otol. (2016) 130:S208–11. 10.1017/S0022215116000645 27841136 PMC4873918

[3] Elbers JB Al-Mamgani A van den Brekel MW Józwiak K de Boer JP Lohuis PJ. Salvage surgery for recurrence after radiotherapy for squamous cell carcinoma of the head and neck. Otolaryngol Head Neck Surg. (2018) 160:1023–33. 10.1177/0194599818818443 30526317

[4] Morton RP Hay KD Macann A. On completion of curative treatment of head and neck cancer: why follow-up? Curr Opin Otolaryngol Head Neck Surg. (2004) 12:142–6. 10.1097/00020840-200404000-00015 15167052

[5] Morris LG Sikora AG Hayes RB Patel SG Ganly I. Anatomic sites at elevated risk of second primary cancer after an index head and neck cancer. Cancer Causes Control. (2011) 22:671–9. 10.1007/s10552-011-9739-2 21327458 PMC3085084

[6] Bugter O van de Ven SE Hardillo JA Bruno MJ Koch AD Baatenburg de Jong RJ. Early detection of esophageal second primary tumors using Lugol chromoendoscopy in patients with head and neck cancer: a systematic review and meta-analysis. Head Neck. (2019) 41:1122–30. 10.1002/hed.25548 30593712 PMC6590301

[7] Funk GF Karnell LH Christensen AJ. Long-term health-related quality of life in survivors of head and neck cancer. Arch Otolaryngol Head Neck Surg. (2012) 138:123–33. 10.1001/archoto.2011.234 22248560

[8] Jansen F Verdonck-de Leeuw IM Cuijpers P Leemans CR Waterboer T Pawlita M. Depressive symptoms in relation to overall survival in people with head and neck cancer: a longitudinal cohort study. Psychooncology. (2018) 27:2245–56. 10.1002/pon.4816 29927013 PMC6231089

[9] Rieke K Schmid KK Lydiatt W Houfek J Boilesen E Watanabe-Galloway S. Depression and survival in head and neck cancer patients. Oral Oncol. (2017) 65:76–82. 10.1016/j.oraloncology.2016.12.014 28109472 PMC8201663

[10] Haeggblom L Attoff T Yu J Holzhauser S Vlastos A Mirzae L. Changes in incidence and prevalence of human papillomavirus in tonsillar and base of tongue cancer during 2000-2016 in the Stockholm region and Sweden. Head Neck. (2018) 41:1583–90. 10.1002/hed.25585 30584688

[11] Wang MB Liu IY Gornbein JA Nguyen CT. HPV-positive oropharyngeal carcinoma: a systematic review of treatment and prognosis. Otolaryngol Head Neck Surg. (2015) 153:758–69. 10.1177/0194599815592157 26124261

[12] Alias A Henry M. Psychosocial effects of head and neck cancer. Oral Maxillofac Surg Clin North Am. (2018) 30:499–512. 10.1016/j.coms.2018.06.010 30266192

[13] Goldsmith T Jacobson MC. Managing the late effects of chemoradiation on swallowing: bolstering the beginning, minding the middle, and cocreating the end. Curr Opin Otolaryngol Head Neck Surg. (2018) 26:180–7. 10.1097/MOO.0000000000000455 29708903

[14] Giannitto C Preda L Zurlo V Funicelli L Ansarin M Di Pietro S. Swallowing disorders after oral cavity and pharyngolaryngeal surgery and role of imaging. Gastroenterol Res Pract. (2017) 2017:7592034. 10.1155/2017/7592034 28496456 PMC5381198

[15] Høxbroe Michaelsen S Grønhøj C Høxbroe Michaelsen J Friborg J von Buchwald C. Quality of life in survivors of oropharyngeal cancer: a systematic review and meta-analysis of 1366 patients. Eur J Cancer. (2017) 78:91–102. 10.1016/j.ejca.2017.03.006 28431302

[16] Heijnen BJ Speyer R Kertscher B Cordier R Koetsenruijter KW Swan K. Dysphagia, speech, voice, and trismus following radiotherapy and/or chemotherapy in patients with head and neck carcinoma: review of the literature. Biomed Res Int. (2016) 2016:6086894. 10.1155/2016/6086894 27722170 PMC5045989

[17] Lahtinen S Koivunen P Ala-Kokko T Kaarela O Laurila P Liisanantti JH. Swallowing-related quality of life after free flap surgery due to cancer of the head and neck. Eur Arch Otorhinolaryngol. (2018) 276:821–6. 10.1007/s00405-018-05264-w 30593593 PMC6411665

[18] Agarwal J Palwe V Dutta D Gupta T Laskar SG Budrukkar A. Objective assessment of swallowing function after definitive concurrent (chemo)radiotherapy in patients with head and neck cancer. Dysphagia. (2011) 26:399–406. 10.1007/s00455-011-9326-4 21344191

[19] Wang X Eisbruch A. IMRT for head and neck cancer: reducing xerostomia and dysphagia. J Radiat Res. (2016) 57 (Suppl. 1):i69–75. 10.1093/jrr/rrw047 27538846 PMC4990117

[20] Strojan P Hutcheson KA Eisbruch A Beitler JJ Langendijk JA Lee AW. Treatment of late sequelae after radiotherapy for head and neck cancer. Cancer Treat Rev. (2017) 59:79–92. 10.1016/j.ctrv.2017.07.003 28759822 PMC5902026

[21] Deutschmann MW McDonough A Dort JC Dort E Nakoneshny S Matthews TW. Fiber-optic endoscopic evaluation of swallowing (FEES): predictor of swallowing-related complications in the head and neck cancer population. Head Neck. (2013) 35:974–9. 10.1002/hed.23066 22730220

[22] Eisbruch A Lyden T Bradford CR Dawson LA Haxer MJ Miller AE. Objective assessment of swallowing dysfunction and aspiration after radiation concurrent with chemotherapy for head-and-neck cancer. Int J Radiat Oncol Biol Phys. (2002) 53:23–8. 10.1016/S0360-3016(02)02712-8 12007937

[23] Raguse J-D Hossamo J Tinhofer I Hoffmeister B Budach V Jamil B. Patient and treatment-related risk factors for osteoradionecrosis of the jaw in patients with head and neck cancer. Oral Surg Oral Med Oral Pathol Oral Radiol. (2016) 121:215-21.e1. 10.1016/j.oooo.2015.10.006 26703417

[24] Lien C-F Wang C-C Hwang T-Z Liu C-F Lin B-S Weng H-H. Comparison between open partial laryngectomy with tube-free tracheostomy and total laryngectomy for hypopharyngeal cancer with cartilage invasion. Oncol Lett. (2018) 16:4961–9. 10.3892/ol.2018.9298 30250561 PMC6144867

[25] Bindewald J Herrmann E Dietz A Wulke C Meister EF Wollbrück D Quality of life and voice intelligibility in laryngeal cancer patients–relevance of the “satisfaction paradox.” Laryngorhinootologie. (2007) 86:426–30. 10.1055/s-2007-966167 17654777

[26] Seiferlein E Haderlein T Schuster M Gräßel E Bohr C. Correlation between coping strategies and subjective assessment of the voice-related quality of life of patients after resection of T1 and T2 laryngeal tumours. Eur Arch Otorhinolaryngol. (2012) 269:2091–6. 10.1007/s00405-012-2020-9 22539095

[27] Davies-Husband C Murphy J Kelly C Drinnan M Paleri V. Extreme long-term voice outcomes after concurrent chemoradiotherapy for advanced non-laryngeal head and neck cancer: eight-year post-treatment analysis. Clin Otolaryngol. (2018) 43:1494–9. 10.1111/coa.13204 30066393

[28] Jacobson BH Johnson A Grywalski C Silbergleit A Jacobson G Benninger MS The Voice Handicap Index (VHI). Am J Speech Lang Pathol. (1997) 6:66–70. 10.1044/1058-0360.0603.66

[29] Balaguer M Percodani J Woisard V. The Carcinologic Handicap Index (CHI): a disability self-assessment questionnaire for head and neck cancer patients. Eur Ann Otorhinolaryngol Head Neck Dis. (2017) 134:399–403. 10.1016/j.anorl.2017.06.010 28826665

[30] Ottosson S Laurell G Olsson C. The experience of food, eating and meals following radiotherapy for head and neck cancer: a qualitative study. J Clin Nurs. (2013) 22:1034–43. 10.1111/jocn.12151 23480499

[31] Li L Mok H Jhaveri P Bonnen MD Sikora AG Eissa NT. Anticancer therapy and lung injury: molecular mechanisms. Expert Rev Anticancer Ther. (2018) 18:1041–57. 10.1080/14737140.2018.1500180 29996062 PMC6290681

[32] Okoye CC Bucher J Tatsuoka C Parikh SA Oliveira GH Gibson MK. Cardiovascular risk and prevention in patients with head and neck cancer treated with radiotherapy. Head Neck. (2017) 39:527–32. 10.1002/hed.24646 28032680 PMC5330677

[33] Roerink SH Coolen L Schenning ME Husson O Smit JW Marres HA. High prevalence of self-reported shoulder complaints after thyroid carcinoma surgery. Head Neck. (2017) 39:260–8. 10.1002/hed.24579 27859810

[34] Keszte J Danker H Dietz A Meister E Pabst F Guntinas-Lichius O. Course of psychiatric comorbidity and utilization of mental health care after laryngeal cancer: a prospective cohort study. Eur Arch Otorhinolaryngol. (2017) 274:1591–9. 10.1007/s00405-016-4340-7 27744529

[35] Zebralla V Kaminski K Wichmann G Dietz A Wiegand S Return to work after curative treatment of Head and Neck Cancer. Laryngorhinootologie. (2018) 97:838–45. 10.1055/a-0731-0344 30536282

[36] Neilson KA Pollard AC Boonzaier AM Corry J Castle DJ Mead KR. Psychological distress (depression and anxiety) in people with head and neck cancers. Med J Aust. (2010) 193(5 Suppl.):S48–51. 21542446 10.5694/j.1326-5377.2010.tb03928.x

[37] Krebber A-MH Jansen F Cuijpers P Leemans CR Verdonck-de Leeuw IM. Screening for psychological distress in follow-up care to identify head and neck cancer patients with untreated distress. Support Care Cancer. (2016) 24:2541–8. 10.1007/s00520-015-3053-6 26694718 PMC4846709

[38] Zigmond AS Snaith RP. The hospital anxiety and depression scale. Acta Psychiatr Scand. (1983) 67:361–70. 10.1111/j.1600-0447.1983.tb09716.x 6880820

[39] Buchmann L Conlee J Hunt J Agarwal J White S. Psychosocial distress is prevalent in head and neck cancer patients. Laryngoscope. (2013) 123:1424–9. 10.1002/lary.23886 23553220

[40] Verdonck-de Leeuw IM Bree R de Keizer AL Houffelaar T Cuijpers P van der Linden MH. Computerized prospective screening for high levels of emotional distress in head and neck cancer patients and referral rate to psychosocial care. Oral Oncol. (2009) 45:e129-33. 10.1016/j.oraloncology.2009.01.012 19362038

[41] Söllner W DeVries A Steixner E Lukas P Sprinzl G Rumpold G. How successful are oncologists in identifying patient distress, perceived social support, and need for psychosocial counselling? Br J Cancer. (2001) 84:179–85. 10.1054/bjoc.2000.1545 11161373 PMC2363697

[42] U.S. Department of Health and Human Services FDA Center for Drug Evaluation and Research; U.S. Department of Health and Human Services FDA Center for Biologics Evaluation and Research, U.S. Department of Health and Human Services FDA Center for Devices and Radiological Health. Guidance for industry: patient-reported outcome measures: use in medical product development to support labeling claims: draft guidance Health Qual Life Outcomes. (2006) 4:79 10.1186/1477-7525-4-79 17034633 PMC1629006

[43] Büttner M Zebralla V Dietz A Singer S. Quality of life measurements: any value for clinical practice? Curr Treat Options Oncol. (2017) 18:30. 10.1007/s11864-017-0470-4 28474263

[44] O'Donohoe P Mercieca-Bebber RL Norquist J Chirovsky D Munshi T Tolley C Assessing the comparability of paper and electronic versions of the EORTC QOL module for head and neck cancer: a qualitative study. JMIR Cancer. (2017) 3:e7 10.2196/cancer.7202 28500019 PMC5446668

[45] Duman-Lubberding S van Uden-Kraan CF Jansen F Witte BI Eerenstein SE van Weert S. Durable usage of patient-reported outcome measures in clinical practice to monitor health-related quality of life in head and neck cancer patients. Support Care Cancer. (2017) 25:3775–83. 10.1007/s00520-017-3808-3 28702685 PMC5658458

[46] de Bree R Verdonck-de Leeuw IM Keizer AL Houffelaar A Leemans CR. Touch screen computer-assisted health-related quality of life and distress data collection in head and neck cancer patients. Clin Otolaryngol. (2008) 33:138–42. 10.1111/j.1749-4486.2008.01676.x 18429869

[47] Kisser U Adderson-Kisser C Coenen M Stier-Jarmer M Becker S Sabariego C. The development of an ICF-based clinical guideline and screening tool for the standardized assessment and evaluation of functioning after head and neck cancer treatment. Eur Arch Otorhinolaryngol. (2017) 274:1035–43. 10.1007/s00405-016-4317-6 27695934

[48] Zebralla V Pohle N Singer S Neumuth T Dietz A Stier-Jarmer M. Introduction of the screening tool oncofunction for functional follow-up of head and neck patients. Laryngorhinootologie. (2016) 95:118–24. 10.1055/s-0035-1549858 26190042

[49] Matthijs de Wit L van Uden-Kraan CF Lissenberg-Witte BI Melissant HC Fleuren MA Cuijpers P. Adoption and implementation of a web-based self-management application “Oncokompas” in routine cancer care: a national pilot study. Support Care Cancer. (2018) 27:2911–20. 10.1007/s00520-018-4591-5 30564933 PMC6598735

[50] Peltola MK Lehikoinen JS Sippola LT Saarilahti K Mäkitie AA. A novel digital patient-reported outcome platform for head and neck oncology patients—a pilot study. Clin Med Insights Ear Nose Throat. (2016) 9:1–6. 10.4137/CMENT.S40219 27721662 PMC5040424

[51] Semple CJ Lannon D Qudairat E McCaughan E McCormac R. Development and evaluation of a holistic surgical head and neck cancer post-treatment follow-up clinic using touchscreen technology-Feasibility study. Eur J Cancer Care. (2018) 27:e12809. 10.1111/ecc.12809 29419940

[52] Basch E Deal AM Dueck AC Scher HI Kris MG Hudis C. Overall survival results of a trial assessing patient-reported outcomes for symptom monitoring during routine cancer treatment. JAMA. (2017) 318:197–8. 10.1001/jama.2017.7156 28586821 PMC5817466

[53] Kotronoulas G Kearney N Maguire R Harrow A Di Domenico D Croy S. What is the value of the routine use of patient-reported outcome measures toward improvement of patient outcomes, processes of care, and health service outcomes in cancer care? A systematic review of controlled trials. J Clin Oncol. (2014) 32:1480–501. 10.1200/JCO.2013.53.5948 24711559

[54] Holch P Warrington L Bamforth LC Keding A Ziegler LE Absolom K. Development of an integrated electronic platform for patient self-report and management of adverse events during cancer treatment. Ann Oncol. (2017) 28:2305–11. 10.1093/annonc/mdx317 28911065 PMC5834137

[55] Basch E Snyder C. Overcoming barriers to integrating patient-reported outcomes in clinical practice and electronic health records. Ann Oncol. (2017) 28:2332–3. 10.1093/annonc/mdx506 28961852

